# Histone H2A and Bovine Neutrophil Extracellular Traps Induce Damage of *Besnoitia besnoiti*-Infected Host Endothelial Cells but Fail to Affect Total Parasite Proliferation

**DOI:** 10.3390/biology8040078

**Published:** 2019-10-11

**Authors:** Iván Conejeros, Zahady D. Velásquez, Daniela Grob, Ershun Zhou, Hannah Salecker, Carlos Hermosilla, Anja Taubert

**Affiliations:** Institute of Parasitology, Biomedical Research Center Seltersberg, Justus Liebig University-Giessen, 35392 Giessen, Germany; zahady.velasquez@vetmed.uni-giessen.de (Z.D.V.); daniela.grob@vetmed.uni-giessen.de (D.G.); ershun.zhou@vetmed.uni-giessen.de (E.Z.); hannah.salecker@vetmed.uni-giessen.de (H.S.); carlos.r.hermosilla@vetmed.uni-giessen.de (C.H.); anja.taubert@vetmed.uni-giessen.de (A.T.)

**Keywords:** NETs, BUVEC, PMN, endothelium, innate, DNA, histones, *Besnoitia*

## Abstract

*Besnoitia besnoiti* tachyzoites infect and develop in bovine endothelial cells in vivo and trigger the release of neutrophil extracellular traps (NETs) from bovine polymorphonuclear neutrophils (PMN). The purpose of this study was to analyze if pure *B. besnoiti* tachyzoite-triggered NETs would damage endothelial host cells and subsequently influence intracellular development and proliferation of *B. besnoiti* tachyzoites in primary bovine endothelial cells. For comparison purposes, isolated A23187-induced NETs were also used. Thus, we here evaluated endothelial host cell damage triggered by histone 2A (H2A) and *B. besnoiti* tachyzoite-induced NET preparations and furthermore estimated the effects of PMN floating over *B. besnoiti*-infected endothelium under physiological flow conditions on endothelial host cell viability. Overall, all treatments (H2A, *B. besnoiti-*triggered NETs and floating PMN) induced endothelial cell death of *B. besnoiti*-infected host cells. However, though host cell damage led to significantly altered intracellular parasite development with respect to parasitophorous vacuole diameter and numbers, the total proliferation of the parasite over time was not significantly affected by these treatments thereby denying any direct effect of NETs on intracellular *B. besnoiti* replication.

## 1. Introduction

The obligate intracellular parasite *Besnoitia besnoiti* (*B. besnoiti*) replicates in vivo in endothelium and represents the causal agent of besnoitiosis in cattle. Bovine besnoitiosis has a high impact on animal welfare and cattle production. Since 2010, bovine besnoitiosis has been classified as an emerging disease by the European Food Safety Authority (EFSA). Within the life cycle of *B. besnoiti*, cattle act as intermediate hosts whilst the definitive host, shedding oocysts, is still unknown [1,2,3].

Polymorphonuclear neutrophils (PMN) and endothelium are key players of host innate immune responses both interacting with *B. besnoiti* stages during acute infection [4]. In addition to classical effector mechanisms, such as reactive oxygen species (ROS) production, phagocytosis and degranulation, PMN are able to extrude chromatin structures decorated with granular proteins that are able to ensnare and eventually kill pathogens. These structures were first reported by Brinkmann et al. [5] and named neutrophil extracellular traps (NETs). They are involved in several physiopathological processes and are described as a defense mechanism that is directed against pathogens (bacteria, fungi, parasites) but also induced by soluble mediators (for review on NET inducers and respective mechanisms please refer to [6,7]). In case of apicomplexan parasites, NETs were reported to be released by PMN of different donor origin in response to stages of *B. besnoiti* [4], *Neospora caninum* [8,9], *Toxoplasma gondii* [10,11], *Eimeria bovis* [12,13], *Plasmodium falciparum* [14], and *Cryptosporidium parvum* [15], thereby highlighting the conserved nature of NETs formation throughout parasite and host species. Specifically regarding *B. besnoiti*-triggered NET formation the process is correlated with a simultaneous increase in autophagy, a process that involves AMPK phosphorylation indicating some insights about the controlling mechanisms in *B. besnoiti*-triggered NETs formation [16].

Direct interactions of PMN or NETs with endothelium at physiological conditions have already been reported [17,18,19] and indicated a critical role of PMN in the pathophysiology of endothelium impairment. Nonetheless, few data are still available with respect to NET-derived effects on parasite-infected endothelium in vitro and in vivo. In general, endothelial cells react upon parasite infections by a broad spectrum of immune-related reactions, such as upregulation of adhesion molecules (e.g., ICAM-1, VCAM-1, P-selectin, E-selectin), chemokines and PMN adhesion onto activated endothelium [12,20,21,22,23,24]. With this regard, our previous data showed that *B. besnoiti* infections induced the following early innate immune reactions in primary bovine umbilical endothelial cells (BUVEC): i) Increased gene transcription of adhesion and inflammatory molecules (ICAM-1, CXCL1, CXCL8, CCL5, and COX-2), ii) augmented PMN adhesion to BUVEC layers and iii) release of NETs under physiological flow conditions [20].

PMN-derived NETs affect endothelium by increasing endothelial cell (EC) layer permeability and directly damaging single endothelial cells [25,26]. Additionally, NETs induce the expression of leukocyte adhesion molecules in activated ECs and, consequently, enhance local inflammatory responses [27]. EC damage is mainly explained by transiently increased abundance of proteases/proteins in the microenvironment of vessels. Major NET components that were already proven as inducers of EC damage include histone 2A (H2A) [26]. Core histones are the most abundant proteins on NETs (70% of all NET-associated proteins) and H2A represents the 26.9% of the total NETs protein content [28]. Moreover, differences in cytotoxicity are dependent of the histone type, being H2A, H2B, and H4 individually more cytotoxic than a mixture of histones [29]. In addition, a critical role of histone H4 in lytic cell death of smooth muscle cells and endothelial cells in a mice model of atherosclerosis was reported recently [30]. Altogether, this evidence highlights the importance of NET-derived histones in tissue damage originated by NET-releasing neutrophils.

The aim of the current study was to determine whether bovine PMN and especially *B. besnoiti* tachyzoite-triggered NETs in addition to a major single NET component, such as H2A [27], induce cytotoxicity and damage in ECs and further alter intracellular *B. besnoiti* tachyzoite development in endothelial host cells. The current methods included fluorescence- and confocal microscopy applying static or physiological flow conditions on *B. besnoiti*-infected and non-infected primary bovine umbilical vein endothelial cells (BUVEC). Respective analyses were performed on BUVEC treated with H2A and NET preparations triggered by *B. besnoiti* tachyzoites. For comparison purposes, NETs were also induced by the calcium ionophore and PMN activator A23187 [31,32,33,34,35]. This compound has been successfully used to stimulate PMN and isolate NETs from humans [36].

Current data revealed that *B. besnoiti*-triggered NETs and H2A induced cytotoxicity and damage in *B. besnoiti*-infected bovine endothelial cells. With respect to parasite intracellular development, *B. besnoiti* parasitophorous vacuole (PV) diameter and number per host cell were found diminished in treated BUVEC. However, total tachyzoite proliferation over time was not significantly affected by NET-derived treatments, thereby denying a direct effect of NETs on intracellular *B. besnoiti* replication.

## 2. Materials and Methods

### 2.1. Ethic Statement

This study was conducted in accordance to Justus Liebig University Giessen Animal Care Committee Guidelines. Protocols were approved by the Ethic Commission for Experimental Animal Studies of the Federal State of Hesse (Regierungspräsidium Giessen; A9/2012; JLU-No.521_AZ), and in accordance to European Animal Welfare Legislation (ART13TFEU) and current applicable German Animal Protection Laws.

### 2.2. Primary Host Endothelial Cell Isolation and Maintenance

Primary bovine umbilical vein endothelial cells (BUVEC) were isolated from umbilical cords obtained from calves born by sectio caesarea at the Clinic of Obstetrics, Gynecology and Andrology of Small and Large Animals, Faculty of Veterinary Medicine, Justus Liebig University Giessen, Germany. Umbilical cords were kept at 4 °C in 0.9% HBSS–HEPES buffer (pH 7.4; Gibco, Grand Island, NY, USA) supplemented with 1% penicillin (500 U/mL; Sigma-Aldrich, St. Louis, MO, USA) and streptomycin (500 μg/mL; Sigma-Aldrich) for a maximum of 16 h before use. For the isolation of endothelial cells (EC), 0.025% collagenase type II (Worthington Biochemical Corporation, Lakewood, NJ, USA) suspended in Pucks solution (Gibco) was infused into the lumen of ligated umbilical veins and incubated for 20 min at 37 °C in 5% CO_2_ atmosphere. After gently massaging umbilical veins, the cell suspension was collected in RPMI-1640 medium and supplemented with 1 mL fetal calf serum (FCS, Gibco) in order to inactivate collagenase type II. After two washes (350 × *g*, 12 min, 20 °C), cells were resuspended in complete endothelial cell growth medium (ECGM, PromoCell (Heidelberg, Germany), supplemented with 10% FCS), plated in 25 cm^2^ tissue plastic culture flasks (Greiner Bio-One, Frickenhausen, Germany) and kept at 37 °C in 5% CO_2_ atmosphere until confluency. BUVEC were cultured in modified ECGM medium (EGCM, diluted at 30% in M199 medium, supplemented with 5% FCS and 1% penicillin and streptomycin (Sigma-Aldrich)) with medium changes every 2–3 days. BUVEC cell layers were used for *B. besnoiti* infections after three passages in vitro.

### 2.3. Isolation of Bovine PMN

Healthy adult dairy cows served as blood/PMN donors. Animals were bled by puncture of jugular vein and peripheral blood was collected in heparinized sterile plastic tubes (Kabe Labortechnik, Nümbrecht-Elsenroth, Germany). 20 mL of heparinized blood was mixed with 20 mL sterile PBS supplemented with 0.02% EDTA (Sigma-Aldrich), the mixture was carefully layered on top of 12 mL Biocoll^®^ separating solution (density = 1.077 g/L; Biochrom AG, Berlin, Germany) and centrifuged (800 × *g*, 45 min). After removal of plasma and peripheral blood mononuclear cells (PBMC), cell pellet was suspended in 25 mL bi-distilled water and gently mixed for 40 s to lyse erythrocytes. Osmolarity was rapidly restored by supplementing appropriate volumes of Hanks balanced salt solution (4 mL, HBSS 10×; Biochrom AG). For entire erythrocyte lysis, this step was repeated twice and PMN were later suspended in sterile RPMI 1640 medium supplemented with 1% penicillin and 1% streptomycin (Sigma-Aldrich). Counting of PMN was performed in a Neubauer haemocytometer chamber (Laboroptik, Lancing, UK). Finally, freshly isolated bovine PMN were allowed to rest at 37 °C and 5% CO2 atmosphere for 30 min until further use.

### 2.4. In Vitro Maintenance and Harvesting of B. besnoiti (Strain Bb1Evora04) Tachyzoites

Permanent Madin-Darby bovine kidney (MDBK) cells were used as host cells to maintain *B. besnoiti* tachyzoites (strain Bb1Evora04; parasite passage number undetermined) in vitro. MDBK cell layers were cultured in 75 cm^2^ plastic tissue culture flasks (Greiner Bio-One, Frickenhausen, Germany) containing RPMI 1640 (Sigma-Aldrich) cell culture medium supplemented with 2% FCS (Merck, Darmstadt, Germany), 1% penicillin (500 U/mL) and streptomycin (500 mg/mL) (both Sigma-Aldrich) at 37 °C and 5% CO_2_ atmosphere until confluency. MDBK cell layers were then infected with 2 × 10^6^ vital tachyzoites of *B. besnoiti.* Harvesting of *B. besnoiti* tachyzoites was performed as described previously [4].

### 2.5. Detection of Extracellular DNA and Protein Markers of NETs by Confocal and Fluorescence Microscopy

Bovine PMN were co-cultured with *B. besnoiti* tachyzoites (ratio 1:4) for 3 h (37 °C and 5% CO_2_ atmosphere) on 0.01% poly-_L_-lysine pretreated coverslips (15 mm diameter, Thermo Fisher Scientific, Braunschweig, Germany), fixed by adding 4% paraformaldehyde (Merck) in PBS and stored at 4 °C until further staining.

For NETs visualization, 4′,6-Diamidine-2′-phenylindole (DAPI) was used to stain DNA and anti-histone (clone H11-4, 1:200; Merck Millipore (Darmstadt, Germany), #MAB3422) and anti-NE (AB68672, 1:200, Abcam, Cambridge, UK) antibodies were used to stain specific proteins on ETs structures. Therefore, fixed samples were washed three times with PBS, incubated with permeabilization solution (3% BSA, 0.3% Triton X-100 in PBS) for 60 min at room temperature (RT) and incubated with corresponding primary antibodies diluted in permeabilization solution for overnight at 4 °C. Thereafter, samples were washed thrice with PBS and incubated in secondary antibody solutions (Alexa Fluor 488 goat anti-rabbit IgG #A-11008 or Alexa Fluor 594 goat anti-mouse IgG, #A-11005; both Life Technologies (Eugene, Oregon, USA) 30 min, 1:500 in permeabilization solution, RT). Finally, samples were washed thrice in PBS and mounted in DAPI-containing mounting media (Fluoromount G with DAPI; Thermo Fisher Scientific). Visualization was achieved applying confocal microscopy (Zeiss LSM 710, Oberkochen, Germany). Image processing was carried out with Fiji ImageJ software (NIH, USA (https://imagej.net/Fiji)) using Z-project and merged channel plugins restricted to overall adjustments of brightness and contrast.

### 2.6. Preparation of B. besnoiti Tachyzoite- or A23187-Induced NETs

Isolation of NETs was performed as previously described by Barrientos et al. [36] with some modifications. Briefly, 1.5 × 10^6^ bovine PMN/well were seeded in 12-well culture plates and stimulated either with A23187 (5 µM) or 6 × 10^6^ *B. besnoiti* tachyzoites (= 1:4 PMN:tachyzoites ratio) for 3 h (37 °C, 5% CO_2_). After incubation, the medium was carefully aspirated and wells were washed twice with 1 mL of PBS. Then, 400 µL of *Alu*I (4 U/mL, New England Biolabs, Ipswich, Massachusetts, USA) were added and plates were incubated for 20 min at 37 °C and 5% CO_2_. Thereafter, samples were recovered and centrifuged for 5 min at 300 × *g* to remove cells and debris. NET preparations were immediately stored at −80 °C until further quantification and use.

DNA content of NET preparations was estimated by Quant-iT PicoGreen (Thermo Fisher Scientific). Briefly, 2 µL of each NET sample was mixed with 98 µL of TE buffer (1 M Tris pH 7.4; 0.5 M EDTA pH = 8.0) and incubated for 5 min at room temperature (RT), protected from light. Afterwards, DNA content was quantified in a Varioskan fluorescence automated multiplate reader (Thermo Scientific, USA) applying exposition/emission wavelengths of 480/520 nm, respectively. All DNA measurements were performed in duplicates. A standard λ-DNA curve was used to interpolate the DNA concentration of the samples.

### 2.7. Estimation of NET-, DNA- and Histone 2A (H2A)-Induced Endothelial Cell Death

Three different BUVEC isolates were cultured to 100% confluency on 96- or 24-well plates (Greiner Bio-One, Frickenhausen, Germany) depending on the experiment setting. For H2A-related experiments, cells were treated with 10 or 100 µg H2A/mL for 4 h or 12 h. For DNA and NET preparations experiments cells were treated for 12 h. To control the influence of components of NETs, *B. besnoiti* secreted and excreted substances and to test if a single protein such as BSA can influence the observed results we also tested BSA (incubation for 4 h and 12 h) at the same concentrations used for histone H2A (10 and 200 µg/mL), viable and heat-inactivated *B. besnoiti* tachyzoites and supernatants recovered from *B. besnoiti* cell culture after 3 h of infection, named excretory secretory (E/S) components at two different dilutions. At each time point, the medium was removed and cells were analyzed for cytotoxic effects via live/dead staining (5 μM Sytox Orange^®^ (Thermo Fisher Scientific) diluted in modified ECGM medium, 10 min, RT, in the dark). Fluorescence intensity was estimated at 574/570 nm excitation and emission wavelengths, respectively.

### 2.8. Physiological Flow Condition Experiments

Three different BUVEC isolates were cultured in µ-slide-0.4 Luer chambers (IBIDI^®^, Martinsried, Germany) until confluency. BUVEC layers were infected with *B. besnoiti* tachyzoites 12 h before performing flow condition experiments or left uninfected for controls. Culture plates were mounted on the stage of a motorized inverted microscope (Olympus Microscope IX81, Hamburg, Germany) using a top-stage incubator (IBIDI^®^, Martinsried, Germany) with a controlled atmosphere of 5% CO_2_ and 37 °C. The chambers containing non-infected or *B. besnoiti*-infected BUVEC were connected to a pump flow system using Luer adapters and a constant physiological wall shear stress of 1.0 dyn/cm^2^ was applied (syringe pump sp100i; World Precision Instruments, Friedberg, Germany) for 5 min of perfusion of either pure medium or a solution 5 x 10^6^ PMN/mL. After perfusion, plates were carefully removed from IBIDI^®^ chambers, cells were fixed in 4% paraformaldehyde at RT for 10 min and finally washed thrice with sterile PBS for further staining.

### 2.9. Determination of Endothelial Cell Damage Using Isolectin GS-IB4

PFA-fixed BUVEC layers were treated with blocking/permeabilization solution (PBS with 3% BSA, 0.1% saponin; 1 h, RT). Thereafter, the samples were incubated in isolectin GS-IB4 which binds predominantly to the cell membrane of blood vessel endothelia [37]. Isolectin GS-IB4 conjugated with Alexa Fluor 594 (Molecular Probes, Eugene, Oregon, USA) was used at a concentration of 20 µg/mL, diluted in blocking/permeabilization solution and the samples were stained for 20 min at RT, in a humidified chamber according to Tanaka et al. [38]. Then, samples were washed thrice in PBS and mounted using DAPI-containing mounting medium Fluoromount G^®^ (Thermo Fisher Scientific). Of each sample, five random microscopic images were taken applying identical conditions of exposition, light intensity and compensation (Olympus Microscope IX81). Image processing was carried out by Fiji ImageJ^®^ using merged-channel-plugins restricted to overall adjustment of brightness and contrast. In brief, images were converted to eight bit and greyscale color. A sharpen filter was applied followed by the color threshold selection, selecting min error algorithm, and defined as background the isolectin-negative regions. The lack of isolectin-derived signals in BUVEC layers was defined as EC damage. For calculation, data from isolectin-negative area were divided by those of the total area to obtain the percentage of EC damage in each analyzed image. Overall, data from a total of 15 images (resulting from three different BUVEC isolates, and three different PMN isolations) were included in the calculation. A workflow of the procedure to obtain endothelial damage values is presented in the supplementary material (Supplementary Figure S5).

### 2.10. Determination of B. besnoiti Rosette Number and Parasitophorous Vacuoles (PV) Diameter

Three different BUVEC isolates were included in all experiments. For each experimental condition, five microscopic images of *B. besnoiti*-infected BUVEC and non-treated controls were randomly taken via phase contrast microscopy (Olympus Microscope IX81^®^). The number of *B. besnoiti* rosettes present in the PV of infected host cells were counted and PV sizes reflecting differential stages of tachyzoite replication were measured manually by three independent observers using the Image J^®^ software (NIH).

### 2.11. Quantification of B. besnoiti Tachyzoites by qPCR

The number of free-released *B. besnoiti* tachyzoites (extracellular tachyzoites in cell culture supernatants) was determined by qPCR using primers described previously by Cortes et al. [39].

### 2.12. Graphical Representation of Results and Statistical Analyses

Statistical significance was defined by a *p*-value < 0.05. If not otherwise stated, *p*-value was determined using the Kruskal-Wallis test followed by Dunn’s multiple comparisons test. Data are presented as bar graphs (showing mean ± SD) or box-whiskers graphs (showing middle line at median and lines at maximum and minimum values). Capital letter N is used to denote the number of biological replicates (BUVEC isolates) and minuscule letter n is used when the analysis referred to *B. besnoiti* rosettes numbers. All statistical analyses and graphs were performed via the Graph Pad^®^ v. 7.03 software (San Diego, CA, USA).

## 3. Results

### 3.1. B. besnoiti Tachyzoites Induce Bovine NETs

**Figure 1:**
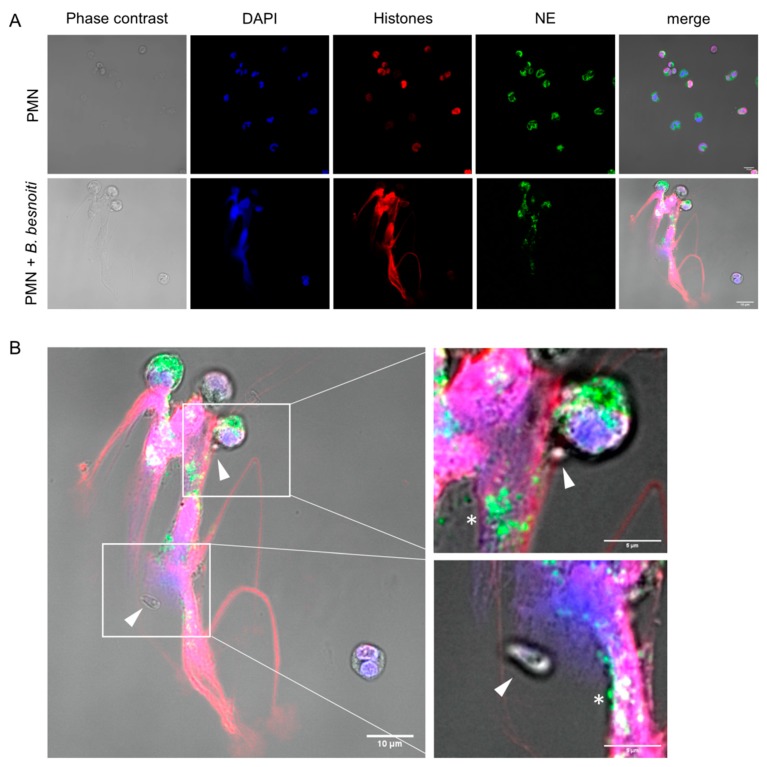
*B. besnoiti* tachyzoites induce neutrophil extracellular traps (NETs). 2 × 10^5^ bovine polymorphonuclear neutrophils (PMN) were confronted with plain medium (control) or 8 × 10^5^ *B. besnoiti* tachyzoites (1:4 ratio) for 3 h at 37 °C and 5% CO_2_. Samples were fixed and immune-stained for neutrophil elastase (green) and histones (red). The coverslips were mounted in mounting media containing 4′,6-Diamidine-2′-phenylindole DAPI to stain DNA (blue). Representative image of NETs in response to *B. besnoiti* tachyzoites is shown in (**A**). In (**B**) a zoom shows the detail of NETs trapping *B. besnoiti* tachyzoites (white triangle). In addition, the dotted distribution of neutrophil elastase in NETs is observed (white asterisk).

Markers of NETs of granular origin, such as neutrophil elastase, were detected colocalizing with histone and DNA when bovine PMN were confronted with *B. besnoiti* tachyzoites at 1:4 ratio for 3 h and observed under confocal microscopy after immunostaining (Figure 1A) or after DNA staining with Sytox Orange (Supplementary Figure S4). Tachyzoites being trapped in NETs were also observed (white triangles) alongside a pointed distribution of neutrophil elastase in NETs (Figure 1B). Images are representative for the experiments conducted with PMN from three different animals. Colocalization of extracellular DNA with these proteins confirmed typical characteristics of NETs. Using the NETs quantification method proposed by González et al. [40] it was estimated that 15% of bovine PMN that were confronted with *B. besnoiti* tachyzoites release NETs (Supplementary Figure S2).

### 3.2. H2A, B. besnoiti Tachyzoite- and A23187-Derived NET Preparations Are All Cytotoxic for BUVEC

**Figure 2:**
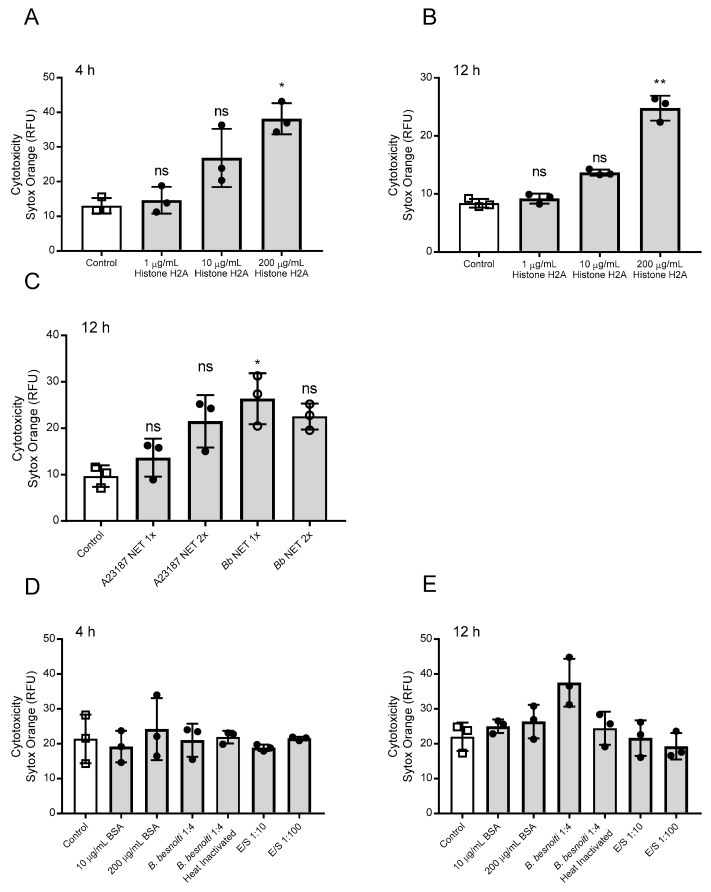
Histone 2A (H2A) and NETs preparations induce cell death in bovine umbilical vein endothelial cells (BUVEC). Three different BUVEC isolates were treated with H2A at concentrations of 1–200 µg/mL for 4 (**A**) h and 12 (**B**) h and with NETs preparations from bovine PMN confronted with *B. besnoiti* tachyzoites (*Bb* NET) or stimulated with A23187 (A23187 NET) for 12 h (**C**). For control purposes BUVEC were incubated with bovine serum albumin (BSA), heat killed, and viable *B. besnoiti* tachyzoites and excretory/secretory (E/S) molecules for 4 h (**D**) and 12 (**E**) h. Cell death was evaluated using Sytox Orange^®^ staining. Bars represent mean ± SD. Statistical significance (ns = non-significant, * *p* < 0.05; ** *p* < 0.01; *p* < 0.001) was determined by Kruskal-Wallis test followed by a Dunn’s post-test comparing experimental versus control conditions (N = 3), all experiments were performed in duplicates.

Cell death was determined by a live/dead-staining with Sytox Orange^®^ (Thermo Fisher Scientific, Waltham, MA USA), which only enters into cells with compromised membranes (= dead cells). Overall, treatments of BUVEC with H2A at 200 µg/mL resulted in significantly enhanced cell death when applied for 4 h (H2A 200 µg/mL versus nontreated control: p = 0.04; Figure 2A) or 12 h (H2A 200 µg/mL versus non-treated control: p = 0.01; Figure 2B). In addition, we tested if pure NET preparations obtained from bovine PMN being stimulated either with *B. besnoiti* tachyzoites (Bb-NETs) or the calcium ionophore A23187 (A23187-NETs) also induced BUVEC death in a static system. In this context, A23187 induced NETs in the bovine system in 39.5% of the cells at the working concentration of 5 µM (Supplementary Figure S1). Preparation of pure NETs resulted in concentrations of 161.5 ± 35 ng DNA/mL for A23187-NETs and 169 ± 17 ng DNA/mL for Bb-NETs. Overall, an average of 40 ng DNA per 10^6^ PMN was obtained. For the estimation of cytotoxic effects on BUVEC, NETs were used at two different concentrations defined as 1X and 2X corresponding to a final concentration of 3.3 and 6.6 ng DNA/mL, respectively. Respective data showed that Bb-NET (1X versus non-treated control: p = 0.03) induced significant cytotoxic effect after 12 h of exposure. On the other hand, both A23187-NET conditions (1X, 2X) showed increased cytotoxicity, but without reaching statistical significance. (Figure 2C). When the influence of protein alone (BSA), *B. besnoiti* tachyzoites (live and heat-killed), and excretory/secretory molecules (E/S) (Figure 2D,E) was analyzed, we found that only viable *B. besnoiti* tachyzoites induced cytotoxicity in BUVEC after 12 h of incubation (Figure 2E).

### 3.3. PMN Induce Endothelial Cell Damage in B. besnoiti-Infected BUVEC Under Flow Conditions

**Figure 3:**
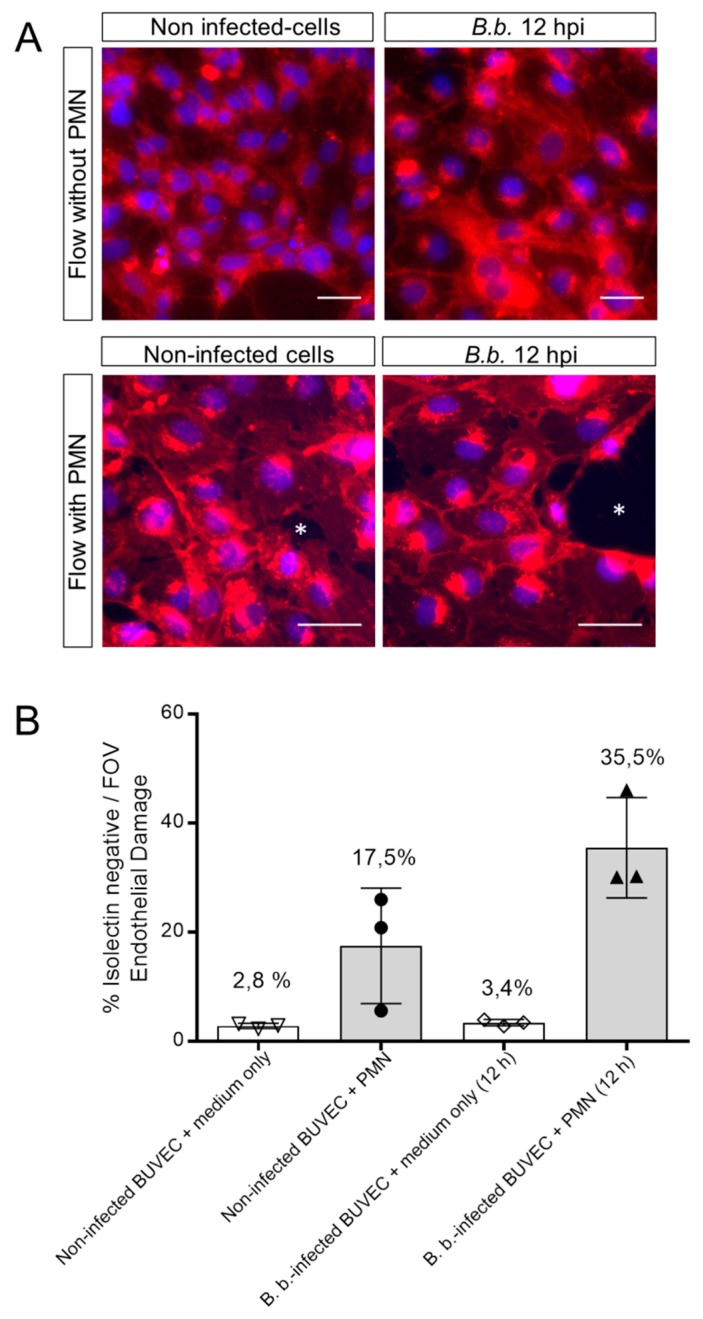
PMN induce damage on *B. besnoiti*-infected BUVEC under physiological flow conditions. PMN or medium alone were perfused at a constant shear stress of 1 dyn/cm^2^ over *B. besnoiti*-infected and non-infected BUVEC at 12 h.p.i. After 5 min of perfusion, cell layers were fixed and stained with DAPI for nuclei and with Alexa Fluor 594-conjugated isolectin-IB4 that predominantly binds endothelium and observed under fluorescence microscopy. Endothelial damage is calculated dividing the isolectin-negative surface (white asterisks, **A**, representative images) by the total surface of the field of view. Column graph represents results of the percentage of endothelial damage after analyzing five random pictures from three different BUVEC isolates per each experimental condition (**B**). FOV = Field of view. Number over the bars indicates the mean % and error bars ± SD.

We recently showed that PMN being perfused over *B. besnoiti*-infected BUVEC show increased endothelial adhesion and additionally form NETs [2]. Here, we applied a constant physiological shear stress of 1 dyn/cm^2^ onto BUVEC and additionally conducted the experiments under controlled temperature (37 °C) and atmosphere (5% CO_2_) conditions. Medium was perfused over BUVEC layers infected with *B. besnoiti* tachyzoites for 12 h for a time period of 5 min under presence or absence of floating bovine PMN. Non-infected BUVEC were used as controls. Following perfusion, BUVEC layers were assayed for the EC damage marker isolectin-IB4 coupled to Alexa Fluor 594 and stained with DAPI as nuclear marker (exemplary images are illustrated in Figure 3A). Unmerged and phase contrast images confirming PMN adhesion on BUVEC layers are presented in supplementary material (Supplementary Figure S3). Overall, perfusion of PMN over non-infected BUVEC induced EC damage (17.48% ± 10.58%), whilst perfusion of medium alone hardly affected BUVEC layers (2.83% ± 0.47%) (Figure 3B). Interestingly, when PMN were perfused over *B. besnoiti*-infected cell layers, EC damage increased to 35.47% ± 9.20% at 12 h of infection (Figure 3B). Given that perfusion of medium alone did not induce considerable EC damage in *B. besnoiti*-infected BUVEC (3.43% ± 0.57% Figure 3B), the former effect could not be due to an enhanced sensitivity of infected BUVEC towards shear stress conditions. Consequently, medium only-related data on non-infected and *B. besnoiti*-infected cells did not differ significantly.

### 3.4. H2A Treatments Decrease PV Diameter in B. besnoiti-Infected Host Cells but Does Not Affect the Number of Rosettes and Total Tachyzoite Production Over Time

**Figure 4:**
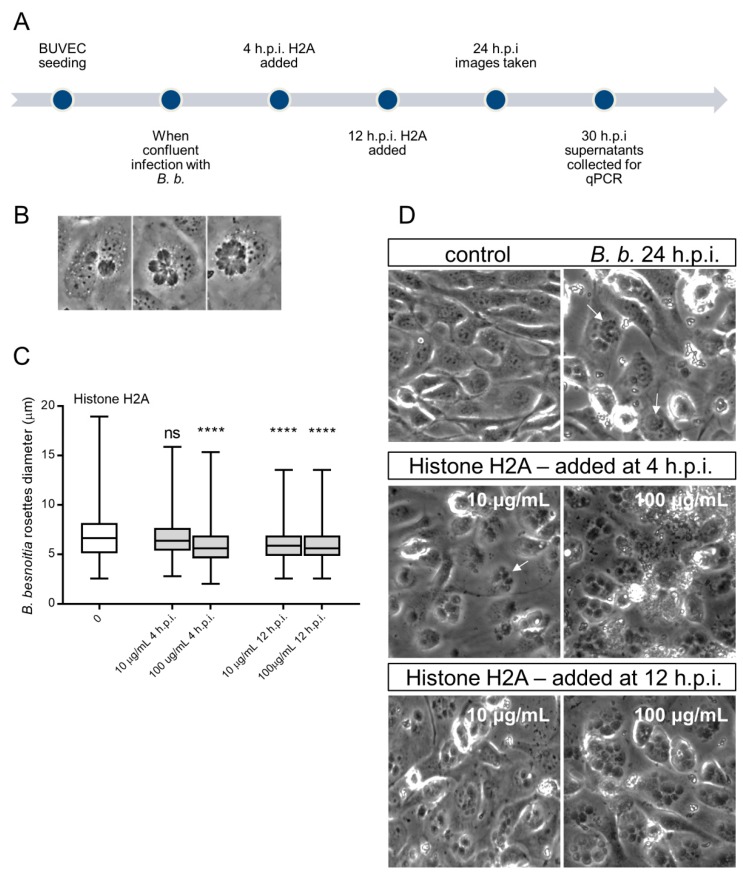
Histone 2A (H2A) treatment of *B. besnoiti*-infected BUVEC reduces *B. besnoiti* parasitophorous vacuole (PV) diameter. BUVEC (three different isolates, N = 3) were treated with H2A at 10 or 100 µg/mL at 4 and 12 h.p.i. (for experimental procedure refer to Figure 4**A**). At 24 h.p.i. experimental conditions were documented by five randomly taken images using a phase contrast microscope (**B**,**D**). (**B**) Shows the typical development of *B. besnoiti* rosettes within 24 h of infection (non-synchronous tachyzoite division leads to the formation of 2-mers to 16-mers). Here, the diameter of each *B. besnoiti* PV was measured (*n* = 825) and plotted as box and whiskers plot (**C**), line at median, bars indicating maximum and minimum values. Statistical significance, N = 3 (ns = non significant, **** *p* < 0.0001) was determined by Kruskal-Wallis test followed by a Dunn´s post-test comparing experimental versus control condition at 4 and 12 h.p.i. In (**D**) a representative image of each experimental condition is shown, white arrows indicate *B. besnoiti* rosettes inside host BUVEC cell.

**Figure 5:**
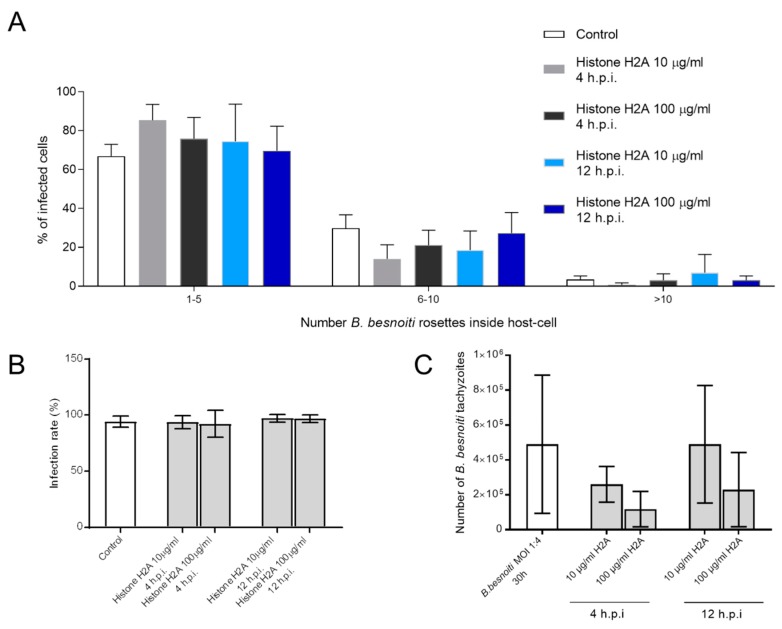
Histone 2A (H2A) did not affect the number of intracellular *B. besnoiti* rosettes. *B. besnoiti*-infected BUVEC (three different isolates, N = 3) were treated with H2A (10 or 100 µg/mL) at 4 and 12 h.p.i. At 24 h.p.i. cell layers were analyzed by randomly taking five microscopic images using phase contrast microscopy. The total number of *B. besnoiti* rosettes per host cell was determined and the percentage of host cells carrying different numbers of PV was estimated and sub-grouped in different categories (with 1–5, 6–10 and >10 rosettes/cell, respectively; (**A**)). Furthermore, the infection rate (**B**) and the total number of tachyzoites being released into cell supernatant within 30 h.p.i. was estimated via qPCR (**C**). Bar graph shows mean ± SD. (*n* = 238).

Since we observed that H2A, NETs and PMN induced cytotoxicity and damage on infected BUVEC under static and physiological flow conditions, we next analyzed whether treatments with H2A as a major component of NETs may also influence intracellular development of *B. besnoiti* (for experimental set-up see Figure 4A). Therefore, we determined *B. besnoiti* PV diameters which reflect the typical division stages of *B. besnoiti* tachyzoites (Figure 4A). In addition, we quantified the number of rosettes present in each host cell. Overall, H2A treatments induced a decrease of the PV diameter independent of the time point of H2A supplementation (Figure 4C). As such, this effect was observed at time points, 4 h and 12 hours post infection (h.p.i.) and at both H2A concentrations (10 and 100 µg/mL) (H2A-treated versus untreated: 100 µg, 4 h: p = <0.0001; 10 µg, 4 h: p = <0.0001; 100 µg, 12 h: p = <0.0001). Referring to mean rosette numbers/host cell, no significant difference was observed between non-treated and H2A-treated samples when varying PV loads per host cell were statistically analyzed within one condition. Given that up to 15 rosettes were detected in non-treated cells, PV number-derived categories were formed and compared to each other. Following this strategy, no changes in mean PV numbers in H2A-treated BUVEC were observed (Figure 5A) whilst the general infection rate remained unchanged (Figure 5B). Accordingly, when estimating tachyzoite production over time (30 h), no significant differences were observed between H2A-treated cells and untreated controls (Figure 5C).

### 3.5. Treatments of B. besnoiti-Infected BUVEC with Bb-NETs and A23187-NETs Affect PV Development and Total Tachyzoite Production

**Figure 6:**
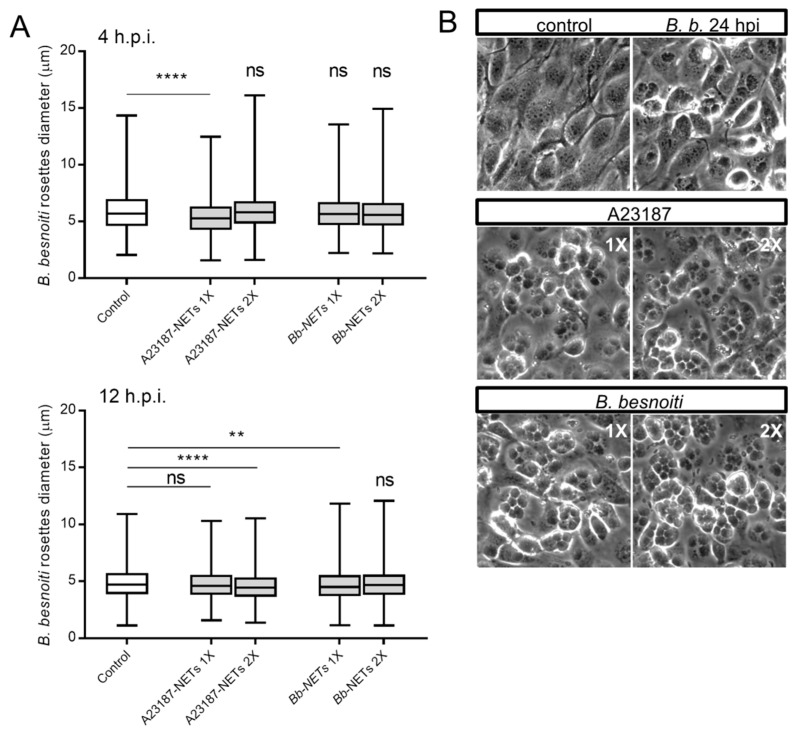
*Bb*-NETs and A231887-NETs induced a decrease in rosette diameter in *B. besnoiti*-infected BUVEC. *Bb*-NETs or A231887-NETs were added at two different concentrations (1X and 2X) to infected BUVEC cells (three different isolates, N = 3) at 4 (**A**, upper panel) and 12 (**A**, lower panel) h.p.i. At 24 h.p.i. experimental conditions were documented within five random pictures using a phase contrast microscope and the diameter of *B. besnoiti* rosettes was determined (*n* = 1115). Box and whiskers plot, line at median, bars indicating maximum and minimum values. Statistical significance was determined by Kruskal-Wallis test followed by a Dunn´s post-test comparing experimental versus control condition at 4 and 12 h.p.i. (**A**). Representative images infected BUVEC cells to which *Bb* and A23187 NETs were added at 12 h.p.i. are shown (**B**). ns = non significant, ** *p* < 0.01, **** *p* < 0.0001.

**Figure 7:**
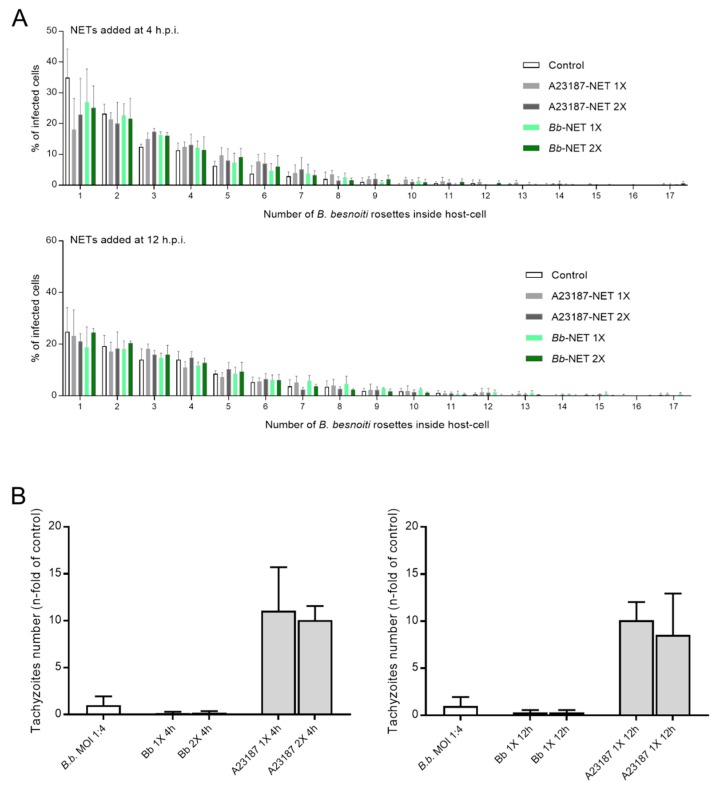
Effects of *Bb*-NET and A23187-NET treatments on intracellular *B. besnoiti* rosettes formation and tachyzoite release. *B. besnoiti*-infected BUVEC (three different isolates, N = 3), were treated with *Bb*-NETs or A231887-NETs at 1X and 2X concentrations at 4 and 12 h.p.i. All treatments were performed in duplicates. At 24 h.p.i. cells were analyzed by randomly taking five images for each experimental condition using phase contrast microscopy. The total number of *B. besnoiti* rosettes per host cell at 4 (*n* = 1115) and 12 h.p.i. (*n* = 970) was determined and the percentage of host cells carrying different numbers of rosettes was calculated (**A**) In addition, the total number of tachyzoites being released into cell supernatant within 30 h.p.i. was estimated via qPCR (**B**). Bars represents mean ± SD.

Overall, treatments of *B. besnoiti*-infected BUVEC layers with Bb-NETs or A23187-NETs at 4 and 12 h.p.i. differentially affected *B. besnoiti* PV diameter. Whilst A23187-NETs induced a significant diminishment of PV size at both time points of treatment (A23187-NETs-1X (4 h) versus untreated control: p < 0.0001; A23187-NETs-1X (12 h) versus untreated control: p = 0.052; A23187-NETs-2X (12 h) versus untreated control: p < 0.0001), treatments with Bb-NETs had no effects when performed at 4 h.p.i. (Figure 6A). However, in the case of later Bb-NET treatments (12 h.p.i.), a significant decrease of PV diameters was estimated for 1X concentration (Bb-NETs-1X (12 h): p = 0.012) (Figure 6A, lower panel). When rosette numbers/host cell were analyzed and normalized as percentage of the infected host cells which contained one to 15 rosettes, we observed a decrease in the number of host cells which contained only one *B. besnoiti* rosette per cell in the case of Bb-NET and A23187-NET treatments (Figure 7A). When estimating total tachyzoite production and release over 30 h, a striking difference was observed when comparing Bb-NET- and A23187-NET-related treatments: Whilst total tachyzoite proliferation was not altered by Bb-NETs (Figure 7B), treatments with A23187-NETs led to a significant and dramatic (almost 10-fold) increase of tachyzoite numbers present in cell culture supernatants (Figure 7B). This effect was independent of both, A23187-NET concentration and the time point of supplementation.

## 4. Discussion

*B. besnoiti* tachyzoites predominantly infect host endothelial cells from different organs and vessels in vivo [20]. In the acute stage, the toxic effect has been related to increased vascular permeability. It is described that these lesions are mainly located in small- and medium-sized vessels, but also in arteries [2]. In this report we used primary endothelial cells isolated from three different animals in order to be as close as possible to the in vivo situation.

We have recently demonstrated that *B. besnoiti* tachyzoites are NET-inducers [4] and that perfusion of bovine PMN over *B. besnoiti*-infected bovine endothelial cells leads to enhanced PMN adhesion and NET deposition on endothelium [20]. NETs are able to entrap B. *besnoiti* tachyzoites thereby hampering the parasite from active host cell invasion. Previously, it was reported that NETs affected tachyzoite-derived host cell invasion since infection rates in primary BUVEC cells decreased by more than 25% when PMN-pre-exposed *B. besnoiti* tachyzoites were used for infection. This effect was reversed by the addition of DNAse, highlighting that NET can potentially affect the continuous infection and proliferation cycles [4]. Additionally, in the acute phase of besnoitiosis leukopenia is observed, an effect that is explained by increased tissue emigration and margination [41]. It is tempting to assume that, as proposed for other intracellular protozoan parasites as *Leishmania* [42], tachyzoite-mediated NETs formation and the corresponding entrapment, induces a series of events involving—at least—mononuclear phagocytes and an inflammatory responses that can restrict the site of the acute infection. Histopathological examination of chronically infected animals has shown inflammatory infiltrates composed by lymphocytes, macrophages, and plasma cells. In some cases, infiltration and disruption of tissue cysts by inflammatory cells was also observed [43]. Adaptive immune reactions of cattle to *B. besnoiti* have been also reviewed [2] and are not covered in this report. Altogether, this evidence gives insights on the multifactorial, complex, and interrelated immune response of cattle to *B. besnoiti* in the acute and chronic stages of the infection.

In this report we confirmed the previous observation and calculated that 15% of PMN release NETs in response to *B. besnoiti* tachyzoites and that the calcium ionophore A23187 induces NETs in the 39.5% and 67.7% of the cells at 5 µM and 25 µM concentration, respectively. On this respect, it must be considered that several NETs quantification systems are described and that the current results must be interpreted in the context of similar methodologies (image analysis) in order to establish correct comparisons [40,44,45]. In addition, we present for the first time evidence that pure NET preparations as well as PMN perfusion under physiological shear stress conditions lead to damage and cell death of parasite-infected endothelial host cells. In addition, we demonstrate that, though PV sizes appear to be affected by NET treatments, NET-related endothelial cell damage fails to significantly influence total parasite proliferation. The current finding emphasizes the hypothesis that excess NET formation may contribute to pathogenesis driven by cell-toxic side-effects and that these immune defence-related structures fail to exert lethal effects on tachyzoite stages.

In the current study, we worked with pure NET preparations that were released from bovine PMN either in response to *B. besnoiti* tachyzoites or after stimulation with the calcium ionophore A23187. In this regard, isolation of NETs was achieved by partial digestion of the DNA backbone by the enzyme nuclease Alu*I* as demonstrated by Barrientos et al. [28]. We here obtained comparable quantities of DNA for both inducers (161.5 ± 35 ng/mL for A23187-NETs and 169.17 ± 17 ng/mL for *Bb*-NETs). These DNA values are one order of magnitude below the ones described for human PMN stimulated with A23187 [36] when normalized as µg of DNA per 1 × 10^6^ PMN. This difference might rely on species-specific differences in the activity of human and bovine PMN [46] and on peculiarities of PMN activation induced by calcium influxes in cattle [32]. No other data exist so far on the recovery or isolation of parasite-induced NETs to conduct a reliable comparison. However, it is expected that NETs derived from *B. besnoiti* tachyzoites and A23187-stimulated PMN contains neutrophil elastase, histones, and MPO considering the detection of these proteins in immunofluorescence of NETs induced by *B. besnoiti* tachyzoites in vitro under static and flow conditions [4,20].

H2A is a key component of NETs and NET-derived H2A was recently reported as a potent inducer of epithelial- and endothelial cell death in both, primary and permanent cell lines [26]. In line, the current data confirmed this effect for primary bovine endothelial cells and additionally showed that cytotoxicity for BUVEC was also observed when cell layers were treated with A23187-NETs and *Bb*-NETs. Considering that histones present in NETs show a lower molecular mass compared to chromatin-derived histones (which may be due to post-translational modifications [28]) and are not as concentrated as when pure H2A is applied, different molecules present in NETs may also have driven these effects. In addition, given that *B. besnoiti*-infected BUVEC generally showed a high infection rate (at least 95% of BUVEC were infected) it additionally appears unlikely that exclusively non-infected cells died within the infected cell layer.

The current study furthermore evaluated endothelial damage under controlled atmosphere and physiological flow conditions by means of the specific marker isolectin IB-4, a lectin derived from *Griffonia simplicifolia* that preferentially binds to endothelium in blood vessels [29], and which was used before in NETs-endothelium damage related studies [38]. The current estimation of the endothelial damage showed that *B. besnoiti*-infected endothelial cells were indeed significantly affected by perfused bovine PMN. Endothelium-PMN interactions, mainly referring to leukocyte adhesion cascades, have extensively been studied comprising a series of steps and signaling pathways [17]. In the case of *B. besnoiti*-infected endothelium, it is known that infection leads to changes in BUVEC host cell metabolism [47]. Moreover, gene expression of the cytokines CXCL1, CXCL8, CCL2, and CCL5 and the adhesion molecules VCAM-1, P-selectin, ICAM-1, and E-selectin was increased in *B. besnoiti*-infected BUVEC at 12 h.p.i. and, in this context, induction of PMN adhesion and NET release occurred under flow [20]. In this study we add evidence that bovine PMN can also induce damage on activated endothelium at the same time point through PMN adhesion and NET release. These lethal effects most probably are due to a transient higher concentration of proteases as hypothesized by others [48].

As an interesting finding, we here showed that the presence of H2A but also of A23187-NETs and *Bb*-NETs led to a decrease of intracellular PV diameter thereby reflecting earlier developmental stages with less numbers of tachyzoites (two- and four-mers of tachyzoites have a smaller diameter than 8- or 16-mers). Noteworthy, this phenomenon occurred irrespective of the time point of *B. besnoiti* infection. The two time points here used, i.e., 4 and 12 h.p.i., reflect *B. besnoiti* replication at the beginning (4 hp.i.: before first division) and in the middle (12 h.p.i.: after two divisions) of first merogony, but before lysis of infected BUVEC occurs (from 20 h ongoing). It must be noted that different *B. besnoiti* strains exhibit different lytic cycle characteristics in vitro when a permanent cell line (MARC-145) is used as a host cell [49]. Our results cannot be compared in terms of the lytic cycle since we use the Bb1Evora04 *B. besnoiti* strain, that was not included in this study and as a host cell we used isolated primary cells (BUVEC). Thus, the timepoint to measure endothelial damage were rationally selected based on the gene expression profile of BUVEC cells infected with *B. besnoiti* tachyzoites and the formation of NETs over infected endothelium [20]. At a first glance, the effects on PV diameter could indicate a direct detrimental effect of H2A or NET preparations on tachyzoite development. However, when host cells were analyzed for rosettes numbers, less cells carrying only one rosette were detected in the case of A23187- and *Bb*-NET treatments. This effect can be linked to selective endothelial cell death, which leads to the release of (obviously) vital tachyzoites which then “rescue” themselves by invading neighboring cells. Since they first have to establish within these new host cells, proliferation onset is delayed, and smaller PVs are found. We consistently observed similar effects in other adverse cell culture conditions, e.g., when cells died out of nutritional deficits. As a general finding, this effect only occurs when single BUVEC die within a total cell layer, not when a cell layer entirely detaches. In agreements with this hypothesis, total *B. besnoiti* tachyzoite production was not significantly affected in NET-treated BUVEC. The latter data clearly indicated that direct lethal effects of NET preparations on intracellular tachyzoites did not occur.

In contrast to H2A or *Bb*-NETs, treatments of *B. besnoiti*-infected BUVEC with A23187-NETs led to a striking and significant increase of *B. besnoiti* tachyzoite production and release into cell culture supernatants within 30 h of infection. The basis of these effects remains unclear although it is known that NET-derived protein composition varies according to the stimuli and may therefore exert different reactions. However, it must be taken into account that existing data mainly refer to PMA-stimulated PMN [28,50], and that also a dependence on the PMN donor species as well as on the quantity of key components present in NETs has been reported [36]. Nevertheless, since Behrendt et al. [51] reported on A23187-induced tachyzoite host cell egress in case of the closely related coccidian parasites *Toxoplasma gondii* and *Neospora caninum*, the increase of free tachyzoites may also have been induced by residues of A23187 compound present in NET preparations (even though these preparations were thoroughly washed before use). However, Behrendt et al. [51] reported that A23187-induced tachyzoite egress largely depends on PV maturity and on A23187 concentration. Thus, exclusively 10 µM A23187 treatments led to egress of tachyzoites from immature PV. Interestingly, reactions were species-dependent since already 1 µM of this compound caused egress of 86% of *T. gondii* tachyzoites but had no effect on *N. caninum* tachyzoites. Given that we here used 5 µM A23197 for NET induction but removed the compound by consecutive washings rather argues against A23187-induced egress as cause for enhanced tachyzoite numbers in cell culture supernatants.

Regarding the mechanisms that can explain our results on, we can hypothesize based on the evidence that shows direct effects of NETs over endothelium: Activation of endothelial pro-MMP-2 and impairment in vasorelaxation by NET through externalization of neutrophil-MMP-9 [52], direct damage to the endothelium by the most prominent histone on NET:H2A; or even degradation products of histones [26,29]. In this context, histones directly activateTLR2 and TLR4 [53] and the corresponding inflammatory signaling cascades [54]. Nevertheless, if these mechanisms are indeed involved in the modulation of intracellular *B. besnoiti* development is unknown so far and may be a matter of further research. Finally, since our data did not include parasite viability or posterior infection rate as parameters is not possible to give a conclusion on this regard.

## 5. Conclusions 

Overall, we here present new data on the damaging capacity of *B. besnoiti* tachyzoite- and A23197-triggered NETs and H2A and showed that PV diameter and number of rosettes/host cell may have been affected by NET-driven host cell death. However, NETs induced by *B. besnoiti* tachyzoites do not influence the total parasite proliferation on infected primary endothelial cells.

## Supplementary Materials

The following are available online at https://www.mdpi.com/2079-7737/8/4/78/s1, Figure S1: Immunofluorescence on PMN releasing NETs in response to A23187 5 and 25 µM and determination of % of PMN releasing NET in these conditions. Figure S2: Estimation of the % of PMN releasing NETs in response to *B. besnoiti* tachyzoites and PMA 100 nM. Figure S3. Additional Images of DAPI/Isolectin IB4 staining of experimental conditions. Figure S4. Additional figures showing *B. besnoiti* tachyzoites inducing bovine NETs. Figure S5. Workflow illustrating the procedure of image analysis to obtain endothelial damage percentages shown in Figure 3.
