# Supplementary material for: Histone H2A and Bovine Neutrophil Extracellular Traps Induce Damage of Besnoitia besnoiti-Infected Host Endothelial Cells but Fail to Affect Total Parasite Proliferation
Source: Biology (Basel). 2019 Oct 11;8(4):78. doi: 10.3390/biology8040078 (PMC6956067; doi:10.3390/biology8040078)
Supplement: Supplementary file 1 [file biology-08-00078-s001.pdf]

Supplementary Material

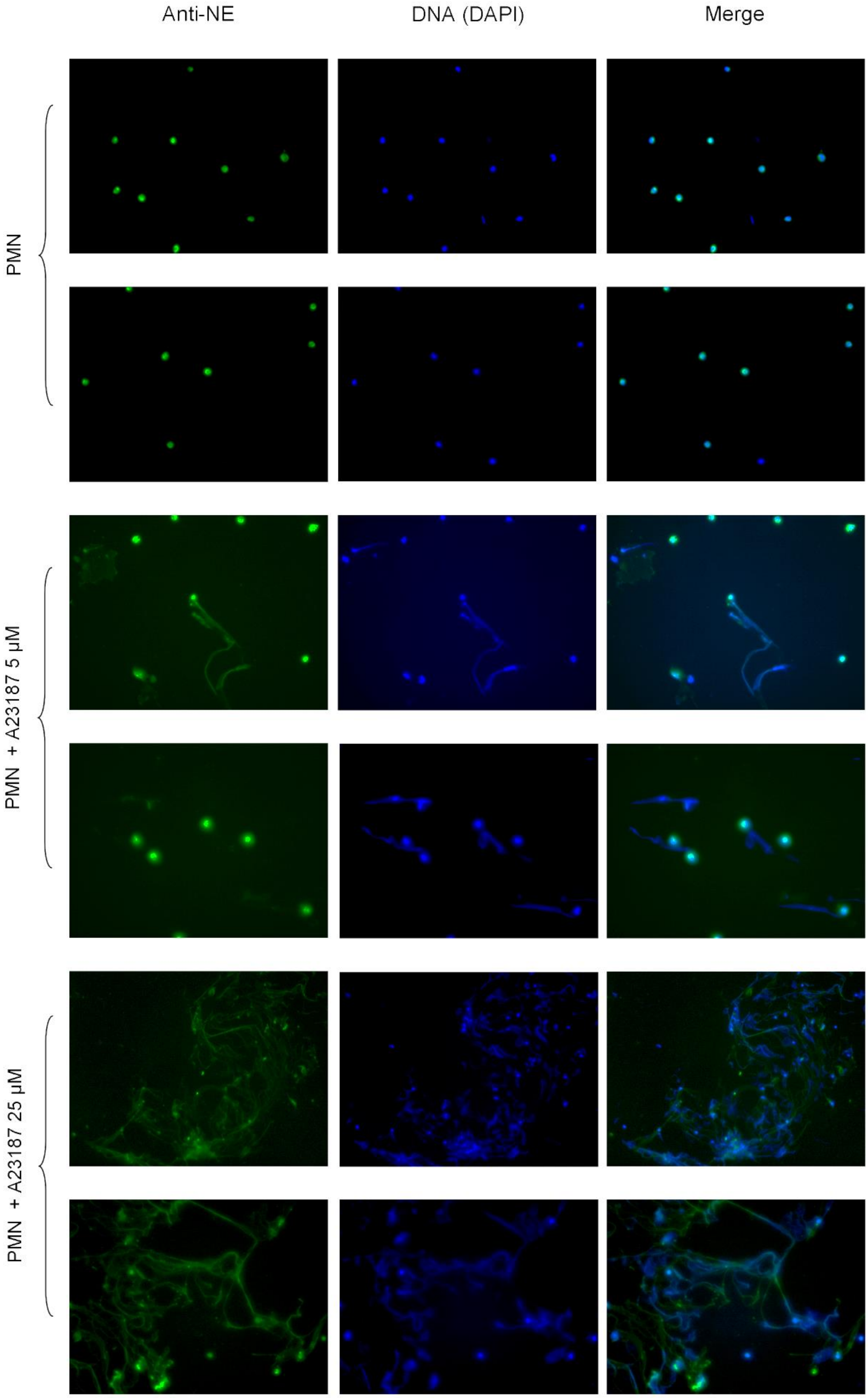

(**A**)

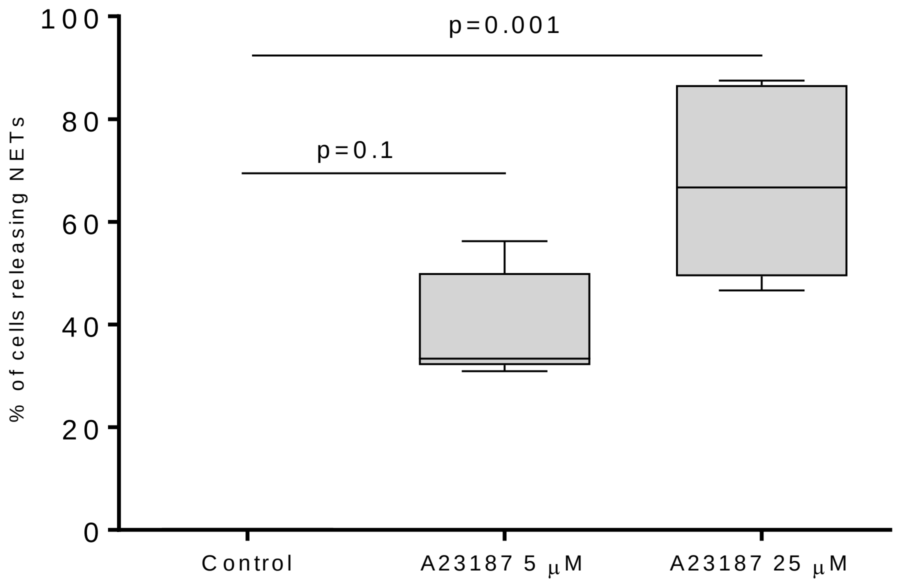

(B)

Figure S1. A23187 induces bovine NETs release. 200.000 bovine PMN were incubated with 5 or 25 μM of A23187 for 3h. Fixed in 4% PFA and treated with specific antibodies to detect Neutrophil elastase. Samples were mounted in DAPI-containing mounting media and the cells releasing NETs were counted. Representative images are shown in (A). Data is represented as box and whiskers plot (B). Statistical significance determined by Kruskal-Wallis and Dunn´s post-test comparing against control condition (PMN + mock).

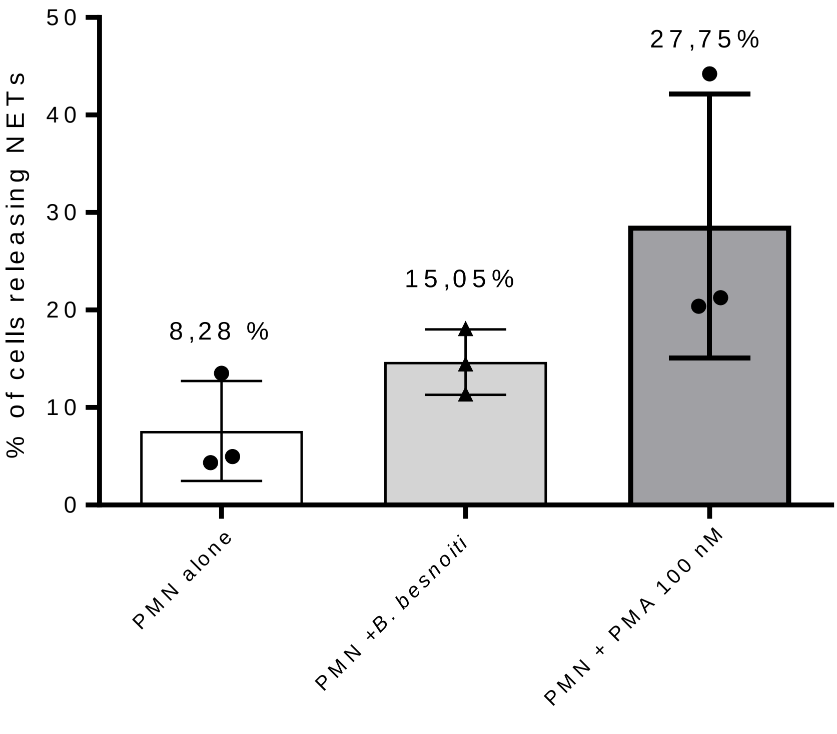

Figure S2. PMN (n = 3) were incubated with *B. besnoiti* tachyzoites, PMA 100 nM or plain media as described in the material and methods section to induce NETs and the % of cells releasing NETs was determined following the methodology proposed by Gonzalez et al. (2014) using Sytox Orange to stain DNA. As suggested, nuclear are expansion over a threshold of 80 μm^2 was considered to

determine a PMN as positive for releasing NETs. Gonzalez, A.S., Bardoel, B.W., Harbort, C.J., Zychlinsky, A., 2014. Induction and quantification of neutrophil extracellular traps. Methods Mol. Biol. 1124, 307–318. https://doi.org/10.1007/978-1-62703-845-4_20

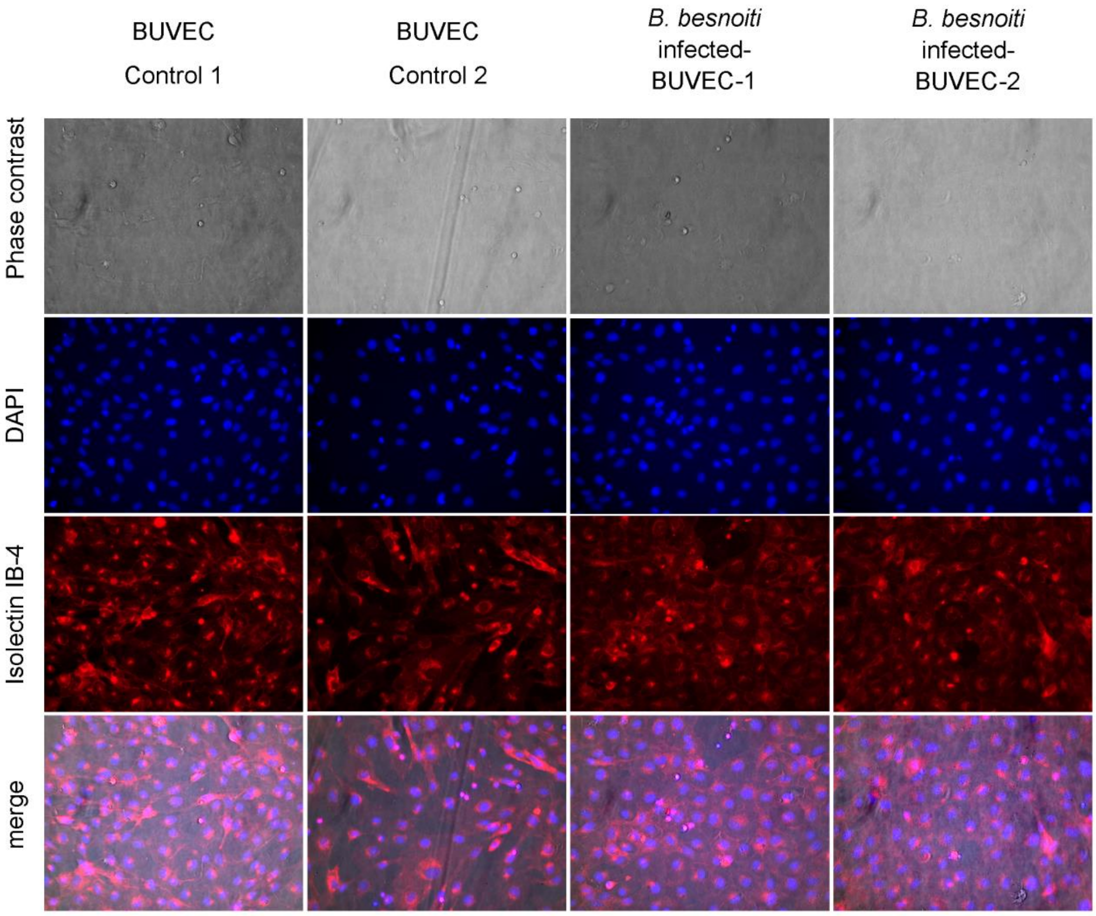

Figure S3. Representative figures obtained in under flow experiments and posterior staining with DAPI (Blue), Isolectin IB-4 coupled with Alexa Fluor 594 (Red). Control and infected conditions are shown. In both cases PMN were perfused at physiological flow (1 dyn/cm^2) before fixing the samples.

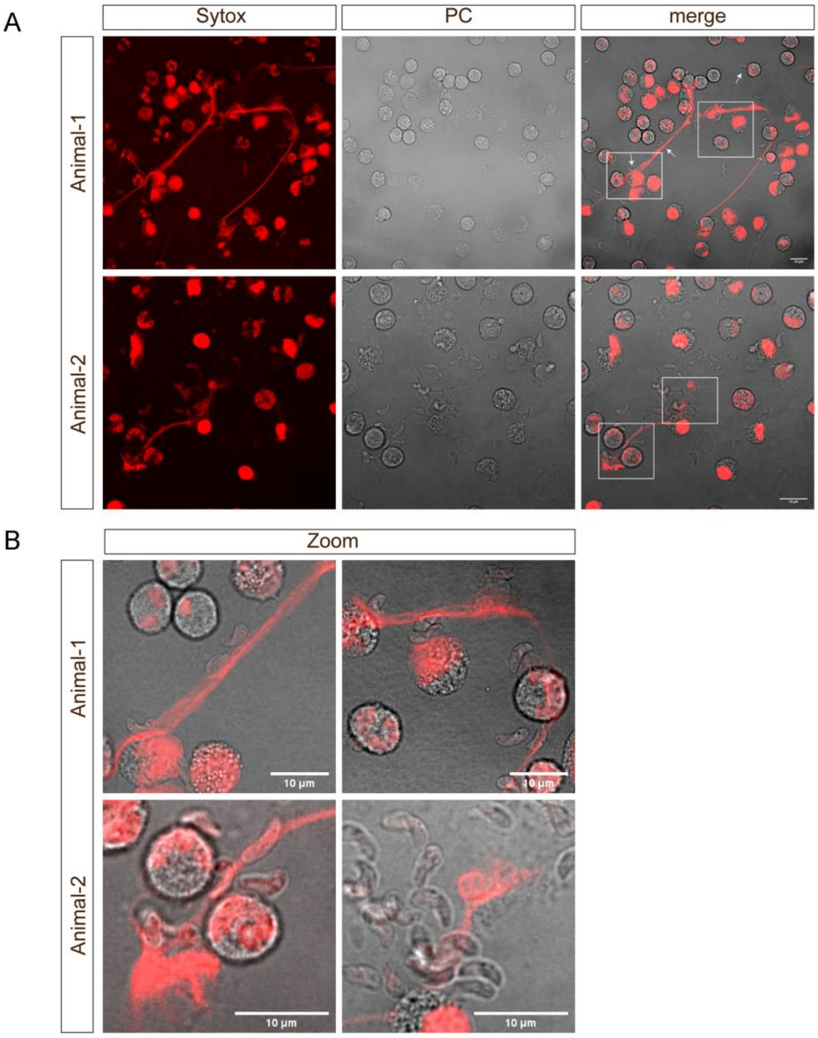

Figure S4. *B. besnoiti* tachyzoites induce NETs. 2 x 10^5 PMN were confronted with 8 x 10^5 *B. besnoiti* tachyzoites (1:4 ratio) for 3 h at 37 °C and 5% CO_2. Samples were fixed and stained for DNA (Sytox Orange). Nuclear decondensation and extracellular DNA entangling *B. besnoiti* tachyzoites were observed under confocal microscopy.

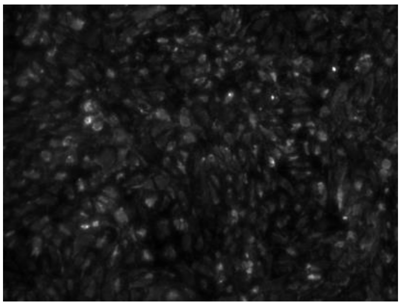

Original Image

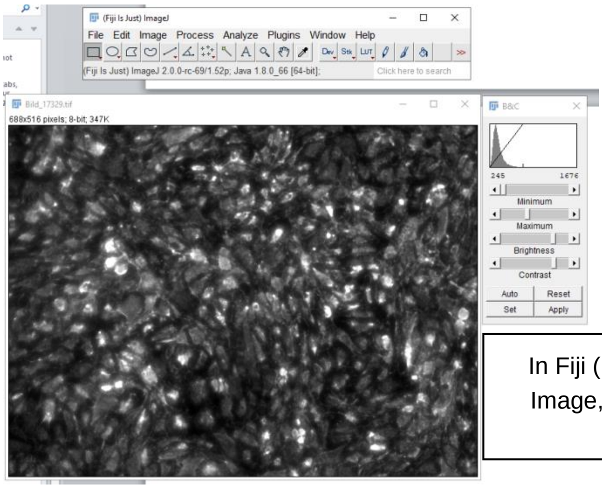

In Fiji (Image J) transform to 8-bit Image, adjust bright and contrast using auto function

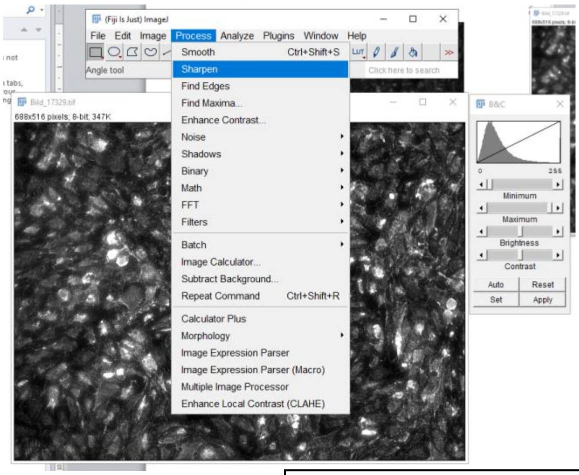

Apply Sharpen filter under the process menu

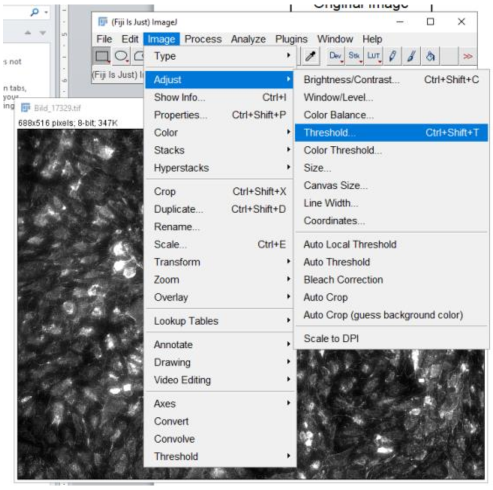

Select Adjust > Threshold under Image menu

Adjust the threshold until all black (negative) regions are considered as a background

Select MinError algorithm of threshold

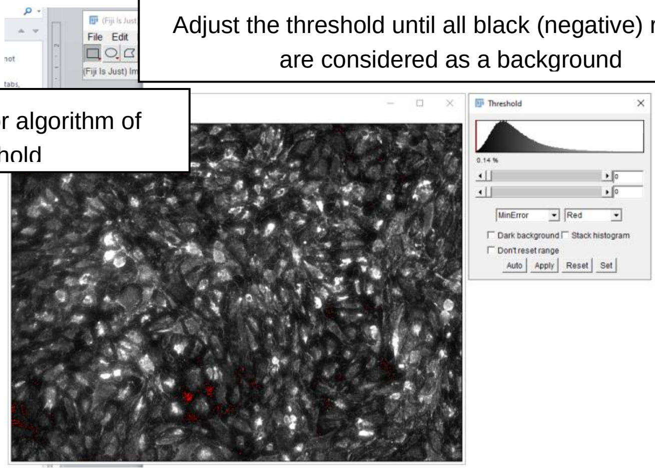

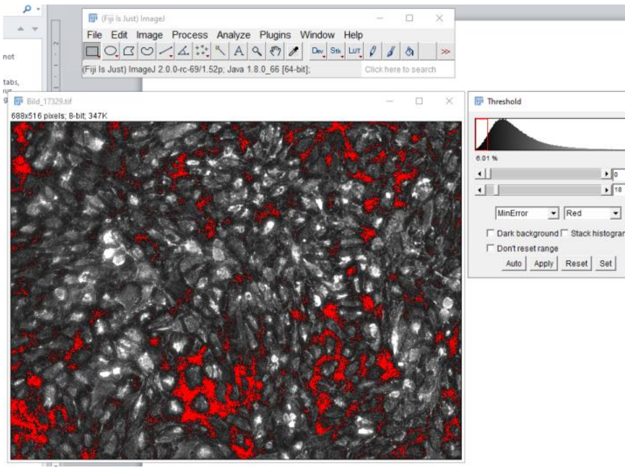

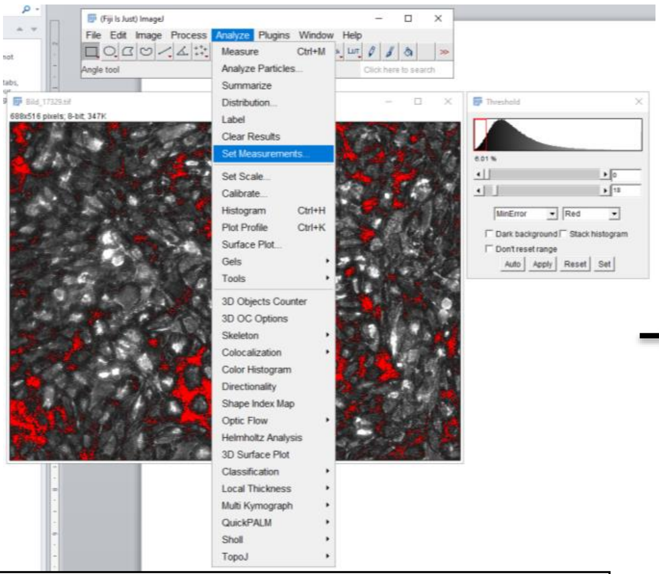

In the analyze menu, select “set measurements”

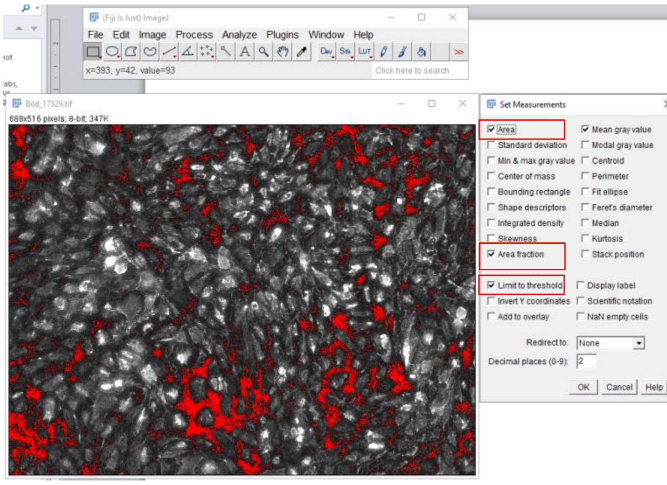

In settings, check Area, limit to threshold and area fraction boxes

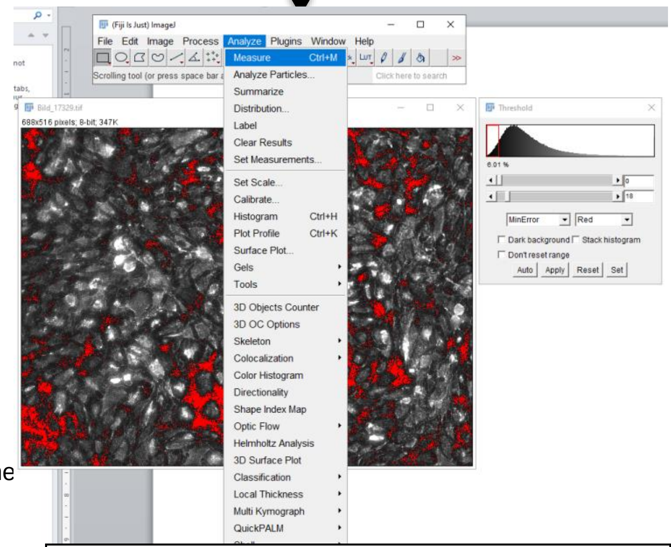

Under Analyze Menu, select Measure

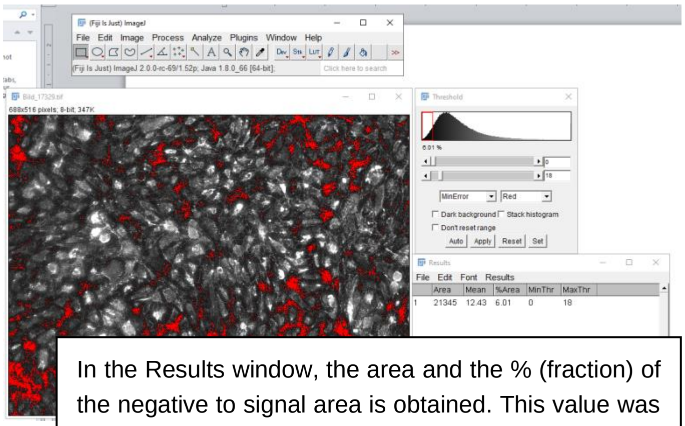

In the Results window, the area and the % (fraction) of the negative to signal area is obtained. This value was considered as “% of endothelial damage” for the figure 3

obtain the
